# Social Capital in Old People Living with HIV Is Associated with Quality of Life: A Cross-Sectional Study in China

**DOI:** 10.1155/2020/7294574

**Published:** 2020-11-30

**Authors:** Ping Lin, Bin Yu, Jiayu Han, Zixin Wang, Peng Jia, Shujuan Yang

**Affiliations:** ^1^Business School, Sichuan University, Chengdu, 610065 Sichuan, China; ^2^West China School of Public Health and West China Fourth Hospital, Sichuan University, Chengdu, 610041 Sichuan, China; ^3^Centre for Health Behaviours Research, The Jockey Club School of Public Health and Primary Care, The Chinese University of Hong Kong, Hong Kong, China; ^4^Department of Land Surveying and Geo-Informatics, The Hong Kong Polytechnic University, Hong Kong, China; ^5^International Institute of Spatial Lifecourse Epidemiology (ISLE), Hong Kong, China

## Abstract

**Objective:**

Old people living with HIV (PLWH) are experiencing a lower quality of life (QoL) than their younger counterparts and have received insufficient attention in China. Given that social capital has been proven to be effective in improving QoL in other countries, we aimed to examine the association between social capital and QoL among old PLWH in China.

**Methods:**

The data presented in this study was based on the baseline sample of an ongoing observational prospective cohort study, which was carried out from November 2018 to February 2019. Participants were old PLWH aged ≥50 in Sichuan, China, and were recruited by stratified multistage cluster sampling from 30 communities/towns. A total of 529 eligible participants finished the face-to-face investigation to measure their social capital (i.e., individual and family- (IF-) based social capital and community and society- (CS-) based social capital) and QoL. The QoL's dimensions of physical health summary (PCS) and mental health summary (MCS) were taken as dependent variables. Stepwise linear regression models were used to examine the association between social capital and QoL.

**Results:**

After considering all significant covariates, the PCS was nonsignificantly correlated with IF-based social capital (*β* = −0.08, 95% CI [-0.28-0.11]) and CS-based social capital (*β* = 0.28, 95% CI [-0.03-0.59]), and MCS was significantly correlated with IF-based social capital (*β* = 0.77, 95% CI [0.54-0.99], *p* < 0.001) and CS-based social capital (*β* = 0.40, 95% CI [0.08-0.72], *p* < 0.05).

**Conclusion:**

Targeted interventions related to building up social capital should be applied to improve the QoL of old PLWH. Providing extra relief funds and allowances might be helpful to improve PCS; improving community networking and engagement and improving family care might be helpful to improve MCS among this vulnerable population.

## 1. Introduction

HIV/AIDS is a serious threat to health for people of all age groups. People living with HIV (PLHW) are faced with the higher risk for some noncommunicable diseases, such as hypertension, diabetes, chronic lung diseases, and cancers (e.g., anal, liver, oral cavity, and pharyngeal cancers) [1, 2], as well as with more serious burdens of some communicable diseases, such as hepatitis B infection. Globally, there were roughly 3,136,500 cases of PLWH in 2017 [3]. Moreover, the PLWH aged over 50 years, also referred to as the old PLWH [4, 5], are more vulnerable to the aforementioned issues than their younger counterparts due to the poor immunity [2, 6]. Also, old PLWH are more susceptible to acquired immunodeficiency syndrome- (AIDS-) related opportunistic infections (e.g., mycobacterium tuberculosis), compared with their younger counterparts [7–9].

The PLWH's quality of life (QoL), usually defined as an individual's overall perception of their physical state, psychological functions, social ability, and individual comprehensive conditions based on social economy, cultural background, and value orientation [10], is generally lower than that of the general population, due to HIV-related disabilities [6] and ongoing widespread stigma [11–13]. The old PLWH, compared to the young PLWH and the general old population, usually face a higher level of stigma [13, 14], a high risk of comorbidity [15], more side effects from antiretroviral therapy (ART) [16], and less social support from friends or families [17]. Recent studies have examined the roles of interpersonal and social factors in the QoL among PLWH in China, revealing that social trust and social connection may be associated with a mental health summary (MCS) score of QoL [18]. A cross-sectional study of social capital in a group of international participants revealed moderate associations between self-reported physical and psychological conditions and total social capital among PLWH [19]. Besides, a systematic review also suggested that social capital-based interventions targeting old people showed favorable and crucial effects on QoL [20]. However, old PLWH face more plight (e.g., discrimination from family and society) than the general old people, and QoL should be deeply watched among this vulnerable population. Given the advances in ART which has made HIV infection increasingly become a chronic and manageable disease [21], it is of great significance and high priority to improve the QoL of PLWH [22, 23].

Social capital, defined as the characteristics of social organizations, such as trust, norms, and networks, is considered a basic driving force of many health-related factors, including QoL [24–27]. Compared to social trust and social connection, social capital can reflect more pictures of overall support from family and friends, not just the macro dimension. Social capital can improve social efficacy by promoting the coordination of social behaviors [27]. A growing number of studies showed that social capital is an important determinant of health-related problems [20, 28]. Currently, only two studies reported that social capital was correlated with QoL among PLWH in China, with one conducted among 283 general PLWH aged over 18 receiving ART in Anhui Province, China, and the other carried out among 261 general PLWH aged over 18 in Southeast China [18, 29]. However, those associations varied by age. For example, the older PLWH were less likely to disclose their HIV serostatus to family, friends, relatives, and health workers [30, 31]. This may cause the old PLWH to feel more isolated and may cause less self-esteem, participation in community activities (e.g., participation in exercise programs), and confidence in personal coping skills, thus leading to different associations between social capital and QoL among old PLWH [32–34]. However, to the best of our knowledge, none of the previous studies focused on the association between social capital and QoL among the old PLWH. There is an urgent need to address this research gap, in order to design effective and efficient social capital-related interventions to improve the QoL among old PLWH.

Given the rapid economic growth and socioeconomic transformation in China, it is necessary to understand the impact of social capital and other broader social factors on the QoL of old PLWH in this context. To achieve health equity across the country, special attention should be given to those vulnerable areas, such as Southwest China, the most HIV-affected regions across the at-risk groups where, for example, the annual average number of new HIV infections in Sichuan and Yunnan had exceeded 3,000 (old HIV infections account for about 18%) from 2004 to 2016 [35, 36]. Using a unique cohort of vulnerable populations in Southwest China, the Sichuan Old HIV-Infected Cohort Study (SOHICS) [37], this study was aimed at investigating the association between social capital and QoL among old PLWH. A previous study has revealed a positive association between social capital and mental health among this population [37]. Moving one step forward, the findings from this study would provide further information for the development of evidence-based interventions and policies for improving the QoL of old PLWH.

## 2. Methods

### 2.1. Study Design, Participants, and Setting

The data presented in this study was based on the baseline sample of the Sichuan Old HIV-Infected Cohort Study (SOHICS), which is an ongoing observational prospective cohort study [37]. The questionnaire used in this study was part of the baseline survey of the cohort cross section conducted from November 2018 to February 2019. The study protocol was approved by the Ethics Committee of the West China School of Public Health and the West China Fourth Hospital, Sichuan University. The study areas of SOHICS were described previously, and a stratified multistage cluster sampling design was applied. A total of 30 communities or towns were finally included into this study [37]. The inclusion criteria for SOHICS participants were as follows: (1) having received a confirmatory HIV diagnosis, (2) aged 50 years or older at the time of diagnosis, (3) having lived in Sichuan for more than five years, and (4) having received care and/or treatment in the local township health centers, which were previously described in detail [37]. The exclusion criteria were those who have a major psychiatric illness (e.g., schizophrenia and bipolar disorder) stated in their medical records in the National AIDS Reporting System or were unable to communicate with the interviewers. Based on the National AIDS Reporting System, the medical staff contacted candidate participants on the basis of the inclusion and exclusion criteria and confirmed their eligibility to participate in the study. A total of 556 eligible old PLWH were contacted, and 529 (95.1%) participants agreed and participated in this study. All participants signed informed consent forms before enrollment.

A self-designed questionnaire was used in this study, which was designed by a panel composed of four epidemiologists, one health psychologist, and two staff members from township health centers. At the beginning, the questionnaire was tested and verified through anonymous face-to-face interviews with 50 participants in the local health center (25 randomly selected from each district/county). Their feedback was used to modify and finalize the questionnaire. Slight changes were made to improve the readability of the questionnaire, and there was no major change (e.g., removal of items) to the questionnaire. Then, using this validated questionnaire, participants were anonymously face-to-face interviewed in private rooms of the same medical center for 20-30 minutes. The researchers asked participants about their sociodemographics, including age group, sex, nationality, place of residence, education attainment, marital status, employment status, and average monthly personal income. Detailed information of old PLWH was collected from the National HIV Surveillance System, including disease-related characteristics (i.e., route of HIV transmission, infection status of the spouse with HIV, time since diagnosis, duration of antiretroviral therapy, and their CD4+ cell counts in the most recent episode of testing).

### 2.2. Measures

#### 2.2.1. Social Capital

The measurement of social capital used in this study was adapted from two scales in a validated Chinese version of the health-related social capital measurement [38], i.e., the individual and family- (IF-) based social capital scale and the community and society- (CS-) based social capital scale. The Social Capital Questionnaire related to the old PLWH had been used in our previous study, and Cronbach's alphas of the IF-based and CS-based social capital scales were 0.638 and 0.657, respectively, which were considered acceptable [37, 39, 40]. The IF-based social capital scale included seven items quantifying participants' relationships and networks with family members, relatives, and neighbors (e.g., you have many close contacts) and was measured by summing up all scores. The CS-based social capital scale was evaluated by seven items, including participation in neighborhood activities, sense of belongingness, social trust, and equity (e.g., you have frequently participated in activities organized by community organizations in the last year). The CS-based social capital scale was also measured by summing up all scores of the seven items. Response categories for both scales used a five-point Likert scale ranging from 1 (strongly disagree) to 5 (strongly agree), with a higher total score exhibiting a stronger social capital (Table S1).

#### 2.2.2. Quality of Life

The Short-Form Health Survey (SF-12) is one of the most widely used instruments for evaluating QoL [16, 17], especially among the PLWH with high reliability and validity [41, 42]. The SF-12 questionnaire includes 12 questions that measure eight health dimensions of the two scales, physical health summary (PCS) and MCS, to assess physical and mental health. The PCS scale includes general health perception, physical functioning, role physical, and body pain; the MCS scale includes vitality, social functioning, role emotional, and mental health. Cronbach's alphas of the PCS and MCS scales were 0.655 and 0.895, respectively. PCS and MCS scores were measured by summing up the scores of their related items and were calculated by *T*-score transformation [43]. Their scores ranged from 0 to 100.

#### 2.2.3. Covariates

Covariates considered in this study included demographic characteristics and disease-related characteristics. The demographic characteristics included age group, sex, nationality, place of residence, education attainment, marital status, employment status, and average monthly personal income. The disease-related characteristics of old PLWH included route of HIV transmission, infection of the spouse with HIV, time since diagnosis, duration of antiretroviral therapy, and CD4+ cell counts in the most recent episode of testing.

### 2.3. Statistical Analyses

Descriptive statistics were used to summarize the characteristics of participants by expressing the results as mean ± standard deviation (SD) for continuous variables that follow the normal distribution, median for continuous variables that do not follow the normal distribution, or percentages for categorical variables. Using QoL as the dependent variable, univariate linear regression models (*t*-tests or ANOVA tests) were first used to examine the association between each covariate and PCS and MCS among old PLWH, and their regression coefficients (*β*) and 95% confidence interval (CI) were estimated. We employed three linear regression models (i.e., model 1, model 2, and model 3) to analyze the association between social capital and QoL. Model 1 included either IF-based social capital or CS-based social capital to conduct the univariate analysis. Model 2 included both IF-based social capital and CS-based social capital and the two core covariates of age and sex. Model 3 included both IF-based social capital and CS-based social capital and all covariates which were marginally significant (*p* < 0.1) in the respective univariate analysis. The *β* and respective 95% CI were obtained, with *p* < 0.05 taken as statistically significant. The subgroup analyses among participants with an HIV-positive spouse and participants with an HIV-negative spouse were employed, respectively. The subanalyses were conducted in the same way as mentioned above. SPSS version 23.0 for Windows (SPSS, Inc., Chicago, IL, United States) was used for data analysis.

## 3. Results

### 3.1. Description of the Participants

Table 1 shows the study participants' sociodemographic characteristics, indicators for HIV disease status, and social capital. The average age of the participants was 63.0 ± 7.2 years, with 70.9% being male and 99.4% being Hans. Of the participants, 77.1% had their permanent residence registered in a rural area, 16.2% were illiterate, 47.1% had only attended primary school, 51.1% were married and living with their spouse, 60.3% were unemployed, 76.6% earned a monthly income of less than 2,000 RMB (about 299 USD), 38.1% had their spouses diagnosed with HIV, and 65.2% had been infected by sexual behaviors with a nonspouse opposite-sex partner. The mean scores of PCS and MCS were 48.5 ± 11.5 and 52.7 ± 11.7, respectively. The mean scores of the IF-based and CS-based social capital scales were 19.2 ± 4.9 and 23.9 ± 3.1, respectively.

### 3.2. Association between Social Capital and QoL

The univariate linear regression model showed that age group, education attainment, employment status, monthly personal income, and route of HIV transmission were correlated with PCS (all *p* values < 0.1). Furthermore, age group, sex, marital status, employment status, route of HIV transmission, infection of the spouse with HIV, time since diagnosis, and duration of antiretroviral therapy were correlated with MCS (all *p* values < 0.1) (Table 2).

When taking PCS as the dependent variable in model 1, IF-based social capital and CS-based social capital were both uncorrelated with PCS. In model 2, we observed that age ≥ 70 showed significant associations with PCS. In model 3, PCS was significantly correlated with age ≥ 70 (*β* = −3.56, 95% CI [-6.51–-0.59]), education level of primary school (*β* = 3.34, 95% CI [0.67-6.01]), education level of junior high school or above (*β* = 5.39, 95% CI [2.43–8.35]), unemployment (*β* = −2.69, 95% CI [-5.14–-0.24]), monthly personal income of 1,000-1,999 RMB (*β* = 5.12, 95% CI [1.38–8.87]), and monthly personal income over 2,000 RMB (*β* = 4.84, 95% CI [0.69–8.99]), but not with IF-based social capital (*β* = −0.08, 95% CI [-0.28–0.11]) and CS-based social capital (*β* = 0.28, 95% CI [-0.03–0.59]) (Table 3).

When taking MCS as the dependent variable in model 1, IF-based social capital and CS-based social capital were both correlated with MCS. In model 2, we observed that IF-based social capital, CS-based social capital, age ≥ 70, and female gender were significantly correlated with MCS. In model 3, MCS was significantly correlated with age ≥ 70 (*β* = 3.30, 95% CI [0.27–6.34]), being married and living with the spouse (*β* = −10.38, 95% CI [-15.17–-5.60]), being married but not living with the spouse (*β* = −9.01, 95% CI [-14.29–-3.73]), being divorced/widowed (*β* = −6.67, 95% CI [-11.74–-1.60]), and IF-based social capital (*β* = 0.77, 95% CI [0.54–0.99], *p* < 0.001) and CS-based social capital (*β* = 0.40, 95% CI [0.08–0.72], *p* < 0.05) (Table 4).

For subgroup analysis among participants with an HIV-positive spouse, both IF-based social capital and CS-based social capital were uncorrelated with PCS, and only IF-based social capital was correlated with MCS in model 3. For subgroup analysis among participants with an HIV-negative spouse, both IF-based social capital and CS-based social capital were uncorrelated with PCS, while both IF-based social capital and CS-based social capital were correlated with MCS in model 3 (Table 5).

## 4. Discussion

This study investigated the association between social capital and QoL in old PLWH, based on the SOHICS baseline in China. Compared to the previous study [18], we focused especially on the old PLWH. After adjusting for demographic characteristics and disease-related characteristics, we found that there remains a negative correlation between the dimensions of social capital (i.e., IF-based social capital and CS-based social capital) and the dimensions of QoL (i.e., MCS). The results support the findings from a cross-sectional study in six countries which demonstrated the relationship between social capital and self-reported health [19] and another study also carried out in Southwest China [18].

Social capital was correlated with MCS in our study. Specifically, a higher score of IF-based social capital and CS-based social capital was correlated with a higher score of MCS, which was consistent with a previous study [18]. There were two possible explanations for this finding. On the one hand, social capital affects the QoL through relieving HIV-related suffering. As previous studies found, social trust and social connection were closely correlated with MCS among PLWH, and social capital was found to decrease the intensity of HIV symptoms, resulting in a greater QoL [18, 44]. On the other hand, a low level of social capital is correlated with an adverse mental status, which might further affect the MCS. As the previous finding showed, the interpersonal networks and the intensity of relationships with family, friends, community, and society play an important role in mental well-being (e.g., anxiety and depression) among old PLWH [37]. Under poor mental status, individuals perceived their poor psychological function, leading to a low MCS status. In China, the staff of community health centers regularly offer ART drugs and psychological counseling during a regular follow-up. As is evident that PLWH under ART in communities showed better QoL than general PLWH [45], those old PLWH under ART usually received accompanying mental care as community social capital, which might further contribute to their improvement of the MCS dimension. However, in our study, all old PLWH were receiving care and/or treatment while showing a relatively low score of CS-based social capital, indicating that the service quality should be improved. Also, standardized practice and equity of mental health services should be considered to effectively reduce disparities in health [46], including QoL, among old PLWH in different regions. Interestingly, according to our subanalysis among participants with HIV-positive spouses, we found that only IF-based social capital was correlated with MCS after adjusting for demographic characteristics and disease-related characteristics. This finding suggested that for both spouses infected with HIV, care from each other and support from family and friends might be more useful to improve their MCS. However, HIV-infected spouses may experience more severe discrimination from family and friends, which needs to be paid attention to in future intervention work [47].

Some demographics and disease-related factors significantly affecting PCS or MCS were of discussion. Age, sex, marital status, educational attachment, personal income, and employment status were found to be correlated with QoL, which is consistent with previous studies [48–50]. Firstly, age was negatively correlated with PCS, which was also reported previously [49]. Conceivably, as the body ages, the incidence of some common diseases of old age (e.g., hypertension, coronary heart diseases, cerebrovascular diseases, and malignant tumors) increases rapidly, and the individual's PCS therefore declines. However, aging was correlated with better MCS, which was also proven in a multinational study [50]. Old people over the age of 70 may be more inclined to adopt positive coping styles and show resilience in treating with illness than people less than the age of 70 [51], which might lead to a higher score of MCS. Secondly, sex and marriage were found as other variables correlated with the QoL, and people with spouses scored lower in MCS than those unmarried. However, a previous study revealed that the differences of QoL between sexes disappeared after controlling for employment and educational attachment [52], which is inconsistent with our research. A possible reason might be that most women in our study were infected within the marriage. Marriage and spouse should be a source of family capital; however, for those PLWH infected within marriage, their relationship with their spouse may be more vulnerable, resulting in a low level of MCS. Besides, for old PLWH whose partners do not know their infection status, the relationship crisis after HIV disclosure [53] and the fear of transmission of HIV to their spouses haunt [54], which might leave old PLWH with depression, anxiety, and therefore a low level of MCS.

Thirdly, we found that a higher education level was correlated with the higher PCS. A possible reason might be that highly educated people are usually equipped with more health knowledge beneficial to their PCS [55]. For those under low educational level, they may obtain a lower PCS score for facing more health risks. As previous studies showed, educational disparities explain the risk for many chronic diseases, such as cardiovascular diseases [56], diabetes [57], and hypertension [58], and are also correlated with the risk for disability [59] and death [60]. It is mentioned that education level is usually related to occupation and income status. Unemployment and low income have also been proven to be positively correlated with a low level of QoL [48, 61]. It is conceivable that those PLWH under unemployment or poor income conditions have poor accessibility of health care services and social support than the general population, which affect their QoL [48]. These findings suggest the importance of improving educational, economic, and employment conditions for PLWH. For the old PLWH in China, most of them were unemployed, and their economic condition is largely dependent on meager allowances and family support (e.g., alimony for children), so their QoL requires special attention.

These findings highlight the importance of social capital for the QoL and provide support that improving interpersonal support, promoting social trust, and health care participation through targeted interventions for PLWH may be effective and evidence-based strategies for improving their QoL [62]. Since the difference of QoL between old PLWH and general PLWH aged less than 50 might be ascribed to the difference in social capital, old PLWH may need more social capital-related interventions and care. In particular in China, where the population is aging at an alarming rate, the old often feel lonely or depressed because of their empty nests [63], which might lead to their low level of MSC. Additionally, old PLWH are less likely to disclose their HIV serostatus to family or friends, which may make these old PLWH feel isolated and is detrimental to their health care and mental care and lead to lower levels of MCS. More interventions related to the improvement of community networking and engagement, family care, and confidence in personal coping skills should be applied to this vulnerable population to improve their MCS. In addition, our results indicate again that an overall measure of “health-related QoL” may not adequately reflect important differences between determinants of physical and mental health and that QoL studies should report results for these domains separately [50]. It is mentioned that nonsignificant results were shown for the domains of PCS, which was inconsistent with a previous study conducted among general PLWH [18]. Other previous study revealed that social capital was closely correlated with enrollment of community-based health insurance [64], through which social capital might show an association with PCS. However, the relief funds and allowance our study population received were generally insufficient (just as our results showing that more than half of the participants received an average monthly personal income less than 1,000 RMB), and this association might be therefore obscured. Demographics and AIDS-related factors significant in the final summary model have led us to think that interventions should be differently targeted for people of different demographics. The results of subgroup analysis among participants with an HIV-positive spouse also led us to think that interventions based on IF social capital should be emphatically carried out in this population.

There are several limitations in this study. First, this is a cross-sectional study, and the causal inference between social capital and QoL should be treated with caution. Second, since the samples represent one province with a high prevalence of AIDS in China, caution should be taken when generalizing the findings to other geographic regions or countries due to sociocultural differences. Likewise, since all participants enrolled in our study were over 50 years old at the time of diagnosis and receiving care and/or treatment in the local township, the findings may not be generalized without caution to the overall PLWH population in China. Third, Cronbach's alpha of those used scales is low. Besides, the participants' answers to the questionnaire were all self-reported and may have been affected by recall bias or social expectation bias. Fourth, some potential confounding variables like smoking, drinking, route of transmission, and perceived discrimination failed to be considered in our study. Future cohort studies include these confounding variables as measures to better analyze the association between social capital and QoL among old PLWH. Lastly, the mechanisms underlying how social capital may influence the QoL remain unknown and need to be explored through follow-ups.

## 5. Conclusions

This study revealed the positive association between two dimensions of social capital and mental health summary (MCS) among old PLWH in Sichuan Province, China. Providing extra relief funds and allowances might be helpful to improve physical health summary (PCS). Improving community networking and engagement and improving family care might be helpful to improve MCS. Further activities and programs should focus on social capital-related interventions, and the government should issue more policies to promote the community participation of old PLWH. The Sichuan Old HIV-Infected Cohort Study (SOHICS) should continue to provide more longitudinal data to better understand the causal association between social capital and quality of life (QoL) in the future.

## Figures and Tables

**Table 1:** Descriptive statistics of the participants (*n* = 529).

	*n* (%)	Mean ± SD
*Sociodemographic characteristics*		
Age group (years)		
50-59	166 (31.4)	
60-69	262 (49.5)	
≥70	101 (19.1)	
Gender		
Male	375 (70.9)	
Female	154 (29.1)	
Nationality		
Han	526 (99.4)	
Minority	3 (0.6)	
Place of residence		
Rural	408 (77.1)	
Urban	121 (22.9)	
Education attainment		
Illiteracy	86 (16.2)	
Primary school	249 (47.1)	
Junior high school or above	194 (36.7)	
Marital status		
Unmarried	29 (5.5)	
Married and living with the spouse	270 (51.1)	
Married but not living with the spouse	78 (14.7)	
Divorced/widowed	152 (28.7)	
Employment status		
Employed	137 (25.9)	
Retired	73 (13.8)	
Unemployed	319 (60.3)	
Average monthly personal income (in RMB)		
None	42 (7.9)	
<1,000	228 (43.2)	
1,000-1,999	135 (25.5)	
≥2,000	124 (23.4)	
*Disease-related characteristics*		
Route of HIV transmission		
Sexual behavior with the spouse	129 (24.4)	
Sexual behavior with a nonspouse opposite-sex partner	345 (65.2)	
Sexual behavior with a same-sex partner	28 (5.3)	
Blood transfusion	27 (5.1)	
Infection of the spouse with HIV		
Yes	202 (38.1)	
No	298 (56.4)	
Do not have a spouse	29 (5.5)	
Time since diagnosis (years)		
<1	156 (29.5)	
1-3	207 (39.1)	
>3	166 (31.4)	
Duration of antiretroviral therapy (years)		
≤2	350 (66.2)	
>2	179 (33.8)	
Stage of HIV infection		
HIV	249 (47.1)	
AIDS	277 (52.4)	
Missing	3 (0.5)	
CD4+ cell counts in the most recent episode of testing (cells/*μ*l)		
<200	145 (27.4)	
200-350	167 (31.6)	
351-500	124 (23.4)	
>500	87 (16.4)	
Missing	6 (1.2)	
*Social capital*		
Individual and family social capital		19.2 ± 4.9
Community and society social capital		23.9 ± 3.1
Overall social capital		43.2 ± 6.3
*Quality of life*		
Physical health summary		48.5 ± 11.5
Mental health summary		52.7 ± 11.7

RMB: renminbi. 1 USD = 6.7 RMB at the time of the investigation.

**Table 2:** Association between covariates and quality of life.

Variables	PCS		MCS	
	Mean ± SD	*t*/*F*(*P*)	Mean ± SD	*t*/*F*(*P*)
*Sociodemographics*				
Age group (year)				
50-59	50.38 ± 10.21		50.77 ± 13.08	
60-69	48.63 ± 10.47		52.90 ± 11.28	
≥70	45.07 ± 13.06	**7.42 (0.001)**	55.15 ± 9.68	**4.59 (0.011)**
Sex				
Male	48.73 ± 10.94		53.93 ± 10.79	
Female	47.92 ± 11.39	0.77 (0.444)	49.57 ± 13.14	**3.64 (<0.001)**
Nationality				
Han	48.49 ± 11.07		52.67 ± 11.70	
Minority	50.33 ± 10.94	-0.29 (0.774)	51.80 ± 8.78	0.13 (0.898)
Place of residence				
Rural	48.30 ± 11.16		52.52 ± 11.64	
Urban	49.15 ± 10.74		53.14 ± 11.84	
Education attainment				
Illiteracy	45.26 ± 12.40		53.46 ± 12.05	
Primary school	47.49 ± 11.34		52.14 ± 11.78	
Junior high school or above	51.22 ± 9.41	**10.99 (<0.001)**	52.98 ± 11.41	0.53 (0.591)
Marital status				
Unmarried	42.89 ± 14.89		56.43 ± 11.40	
Married and living with the spouse	48.76 ± 10.64		52.86 ± 11.21	
Married but not living with the spouse	50.29 ± 10.10		52.86 ± 12.17	
Divorced/widowed	48.14 ± 10.76	1.66 (0.170)	51.99 ± 12.28	**2.13 (0.090)**
Employment status				
Employed	52.29 ± 8.14		53.09 ± 10.41	
Retired	50.16 ± 9.37		55.62 ± 9.59	
Unemployed	46.49 ± 12.01	**14.89 (<0.001)**	51.80 ± 12.51	**3.33 (0.040)**
Average monthly personal income (in RMB)				
None	44.87 ± 11.52		53.13 ± 10.05	
<1,000	45.72 ± 11.77		53.03 ± 12.38	
1,000-1,999	51.12 ± 10.23		51.89 ± 11.22	
≥2,000	51.98 ± 8.54	**13.88 (<0.001)**	52.66 ± 11.44	0.29 (0.830)
*Disease-related characteristics*				
Route of HIV transmission				
Sexual behavior with the spouse	47.62 ± 10.96		50.23 ± 12.43	
Sexual behavior with a nonspouse opposite-sex partner	48.16 ± 11.31		52.98 ± 11.63	
Sexual behavior with a same-sex partner	54.25 ± 6.22		56.11 ± 8.90	
Blood transfusion	51.68 ± 10.64	**3.33 (0.020)**	55.80 ± 9.55	**3.50 (0.020)**
Infection of the spouse with HIV				
Yes	48.48 ± 10.51		51.08 ± 11.95	
No	48.84 ± 11.09		53.23 ± 11.44	
Do not have a spouse	45.08 ± 14.09	-0.66 (0.509)	57.84 ± 10.46	**3.06 (0.002)**
Time since diagnosis (years)				
<1	47.64 ± 11.40		51.93 ± 12.86	
1-3	49.25 ± 11.23		51.66 ± 10.94	
>3	48.36 ± 10.53	0.96 (0.390)	54.60 ± 11.23	**3.39 (0.034)**
Duration of antiretroviral therapy (years)				
≤2	48.59 ± 11.18		51.81 ± 12.09	
>2	48.31 ± 10.86	0.27 (0.770)	54.33 ± 10.68	**2.37 (0.040)**
CD4+ cell counts in the most recent episode of testing (cells/*μ*l)				
<200	48.71 ± 10.86		53.38 ± 10.86	
200-350	47.24 ± 11.98		52.77 ± 12.56	
351-500	49.83 ± 10.40		52.13 ± 11.81	
>500	48.58 ± 10.34	1.01 (0.400)	52.13 ± 11.09	0.29 (0.890)

Boldfaced numbers indicate *p* < 0.1. RMB: renminbi.

**Table 3:** Stepwise regression analysis of association between social capital and PCS.

	PCS		
	*β* (95% CI)		
	Model 1	Model 2	Model 3
Individual and family social capital scale	0.04 (-0.15, 0.24)	0.02 (-0.18, 0.22)	-0.08 (-0.28, 0.11)
Community and society social capital scale	0.06 (-0.25, 0.38)	0.12 (-0.20, 0.44)	0.28 (-0.03, 0.59)
Age group (years)			
50-59		Reference	Reference
60-69		-1.89 (-4.03, 0.25)	0.13 (-2.10, 2.36)
≥70		**-5.69 (-8.45, -2.92)** ^∗∗∗^	**-3.56 (-6.51, -0.59)** ^∗^
Sex			
Male		Reference	Reference
Female		-1.16 (-3.25, 0.93)	-0.29 (-0.33, 2.74)
Education attainment			
Illiteracy			Reference
Primary school			**3.34 (0.67, 6.01)** ^∗^
Junior high school or above			**5.39 (2.43, 8.35)** ^∗∗∗^
Employment status			
Employed			Reference
Retired			-2.24 (-5.65, 1.18)
Unemployed			**-2.69 (-5.14, -0.24)** ^∗^
Average monthly personal income (in RMB)			
None			Reference
<1,000			0.78 (-2.72, 4.29)
1,000-1,999			**5.12 (1.38, 8.87)** ^∗∗^
≥2,000			**4.84 (0.69, 8.99)** ^∗^
Route of HIV transmission			
Sexual behavior with the spouse			Reference
Sexual behavior with a nonspouse opposite-sex partner			-0.44 (-3.65, 2.77)
Sexual behavior with a same-sex partner			0.59 (-4.64, 5.82)
Blood transfusion			1.02 (-3.71, 5.75)

^∗^ *p* < 0.05, ^∗∗^*p* < 0.01, and ^∗∗∗^*p* < 0.001. Boldfaced numbers indicate *p* < 0.05. Model 1: including either IF-based social capital or CS-based social capital as an independent variable. Model 2: including two social capital scales, age, and sex. Model 3: including two social capital scales and covariates marginally significant (*p* < 0.1) in respective univariate analysis (age, sex, education attainment, employment status, monthly personal income, and route of HIV transmission). RMB: renminbi.

**Table 4:** Stepwise regression analysis of association between social capital and MCS.

	MCS		
	*β* (95% CI)		
	Model 1	Model 2	Model 3
Individual and family social capital scale	**0.61 (0.42, 0.81)** ^∗∗∗^	**0.53 (0.34, 0.73)** ^∗∗∗^	**0.77 (0.54, 0.99)** ^∗∗∗^
Community and society social capital scale	**0.74 (0.42, 1.06)** ^∗∗∗^	**0.45 (0.13, 0.78)** ^∗∗^	**0.40 (0.08, 0.72)** ^∗^
Age group (years)			
50-59		Reference	Reference
60-69		1.51 (-0.65, 3.67)	1.89 (-0.36, 4.15)
≥70		**3.12 (0.31, 5.91)** ^∗^	**3.30 (0.27, 6.34)** ^∗^
Sex			
Male		Reference	Reference
Female		**-3.44 (-5.55, -1.33)** ^∗∗^	-2.41 (-5.54, 0.71)
Marital status			
Unmarried			Reference
Married and living with the spouse			**-10.38 (-15.17, -5.60)** ^∗∗∗^
Married but not living with the spouse			**-9.01 (-14.29, -3.73)** ^∗∗^
Divorced/widowed			**-6.67 (-11.74, -1.60)** ^∗^
Employment status			
Employed			Reference
Retired			-0.02 (-3.43, 3.83)
Unemployed			-1.96 (-4.34, 2.72)
Route of HIV transmission			
Sexual behavior with the spouse			Reference
Sexual behavior with a nonspouse opposite-sex partner			-1.12 (-4.75, 2.52)
Sexual behavior with a same-sex partner			2.17 (-3.54, 7.89)
Blood transfusion			0.63 (-4.62, 5.88)
Infection of the spouse with HIV			
Yes			Reference
No			1.47 (-1.17, 4.12)
Time since diagnosis (years)			
<1			Reference
1-3			-0.60 (-2.95, 1.74)
>3			2.73 (-1.35, 6.81)
Duration of antiretroviral therapy (years)			
≤2			Reference
>2			-0.86 (-4.44, 2.72)

^∗^ *p* < 0.05, ^∗∗^*p* < 0.01, and ^∗∗∗^*p* < 0.001. Boldfaced numbers indicate *p* < 0.05. Model 1: including either IF-based social capital or CS-based social capital as an independent variable. Model 2: including two social capital scales, age, and sex. Model 3: including two social capital scales and covariates marginally significant (*p* < 0.1) in respective univariate analysis (age, sex, marital status, employment status, route of HIV transmission, infection of the spouse with HIV, time since diagnosis, and duration of antiretroviral therapy).

**Table 5:** Subgroup analyses based on the spouse's HIV status.

	PCS			MCS		
	*β* (95% CI)			*β* (95% CI)		
Subgroup	Model 1	Model 2	Model 3	Model 1	Model 2	Model 3
Have an HIV-positive spouse (*n* = 202)						
Individual and family social capital scale	-0.15 (-0.46, 0.15)	-0.28 (-0.61, 0.05)	-0.24(-0.54, 0.07)	**0.81 (0.47, 1.14)** ^∗∗∗^	**0.65 (0.30, 1.00)** ^∗∗∗^	**0.64 (0.28, 0.99)** ^∗∗∗^
Community and society social capital scale	0.12 (-0.36, 0.60)	0.21 (-0.29, 0.71)	0.27 (-0.19, 0.73)	**0.90 (0.37, 1.43)** ^∗∗^	0.54 (-0.01, 1.08)	0.50 (-0.04, 1.05)
Have an HIV-negative spouse (*n* = 298)						
Individual and family social capital scale	0.08 (-0.18, 0.34)	0.04 (-0.22, 0.30)	-0.09 (-0.36, 0.17)	**0.70 (0.44, 0.96)** ^∗∗∗^	**0.65 (0.39, 0.91)** ^∗∗∗^	**0.59 (0.34, 0.85)** ^∗∗∗^
Community and society social capital scale	0.10 (-0.31, 0.51)	0.16 (-0.26, 0.57)	0.28 (-0.13, 0.69)	**0.65 (0.23, 1.07)** ^∗∗^	0.39 (-0.03, 0.80)	**0.42 (0.02, 0.83)** ^∗^

^∗^ *p* < 0.05, ^∗∗^*p* < 0.01, and ^∗∗∗^*p* < 0.001. Boldfaced numbers indicate *p* < 0.05. Model 1: including either IF-based social capital or CS-based social capital as an independent variable. Model 2: including two social capital scales, age, and sex. Model 3: including two social capital scales and covariates marginally significant (*p* < 0.1) in respective univariate analysis. For the subgroup of having an HIV-positive spouse, when taking PCS as the dependent variable, the covariates marginally significant (*p* < 0.1) in respective univariate analysis included sex, education attainment, employment status, monthly personal income, and CD4+ cell counts in the most recent episode of testing; when taking MCS as the dependent variable, the covariates marginally significant (*p* < 0.1) in respective univariate analysis included age group, sex, route of HIV transmission, time since diagnosis, and duration of antiretroviral therapy. For the subgroup of having an HIV-negative spouse, when taking PCS as the dependent variable, the covariates marginally significant (*p* < 0.1) in respective univariate analysis included age group, education attainment, employment status, and monthly personal income; when taking MCS as the dependent variable, the covariates marginally significant (*p* < 0.1) in respective univariate analysis included sex, CD4+ cell counts in the most recent episode of testing, and time since diagnosis.

## Data Availability

The data was collected by experienced scientists and health personnel. The datasets used and/or analyzed during the current study are available from the corresponding author on reasonable request.

## Supplementary Materials

Supplementary Materials Table S1: social capital of the participants (*n* = 529).
