# Supplementary material for: Social Capital in Old People Living with HIV Is Associated with Quality of Life: A Cross-Sectional Study in China
Source: Biomed Res Int. 2020 Nov 30;2020:7294574. doi: 10.1155/2020/7294574 (PMC7721488; doi:10.1155/2020/7294574)
Supplement: Supplementary Materials — Table S1: social capital of the participants (n = 529). [file 7294574.f1.docx]

**Table S1. Social capital of the participants (n=529)**

| **Variables** | **N** | **%** |
| --- | --- | --- |
| **Individual and family social capital** (% agree/strongly agree) |  |  |
| You have many close contacts | 170 | 32.1 |
| You have had many social interactions with people other than your family members in the past month | 121 | 22.9 |
| You always trust people who have social interactions with you | 258 | 48.8 |
| You are satisfied with your marriage | 201 | 38.0 |
| You have always received emotional/financial/instrumental support from your spouse | 221 | 41.8 |
| You have always received emotional/financial/instrumental support from your relatives | 314 | 59.4 |
| You have always received emotional/financial/instrumental from your close contacts in the last year | 75 | 14.2 |
| **Total mean scores** | 19.2±4.9 |  |
| **Community and society social capital** (% agree/strongly agree) |  |  |
| You have frequently participated in activities organized by community organizations in the last year | 24 | 4.5 |
| You have always received support from community organizations in the last year | 13 | 2.5 |
| You trust health organizations (i.e., hospitals and centers for diseases control and prevention) very much | 507 | 95.8 |
| You trust community organizations very much | 337 | 63.7 |
| You trust other governmental organizations very much | 493 | 93.2 |
| You agree with the statement that hardworking people will be rewarded by the society | 455 | 86.0 |
| You agree with the statement that talented people will be recognized by the society | 435 | 82.2 |
| **Total mean scores** | 23.9±3.1 |  |
